# Supplementary material for: Exploring mental health symptoms in elite athletes during the COVID-19 pandemic: A systematic review and meta-analysis on sex differences
Source: PLoS One. 2025 Jan 16;20(1):e0314996. doi: 10.1371/journal.pone.0314996 (PMC11737678; doi:10.1371/journal.pone.0314996)
Supplement: S1 Table — (DOCX) [file pone.0314996.s002.docx]

**S1 Table. Database Search Strategy**

| **PubMed (March 31^st^ 2024)** |
| --- |
| ("sex"[MeSH] OR "sex"[TW] OR "sex differences"[TW] OR Male[mesh] OR Male[tw] OR Female[mesh] OR Female[tw] OR Man[tw] OR men[mesh] OR men[tw] OR Woman[tw] OR women[mesh] OR women[tw] OR "gender"[TW])  AND  (College[tw] OR collegiate[tw] OR university[tw] OR semi-professional[tw] OR professional[tw] OR national team[tw] OR Olympic[tw] OR Olympics[tw] OR elite[tw])  AND  (Athletes[mesh] OR Athletes[tw] OR Athlete[tw] OR Sports[mesh] OR sports[tw] OR sport[tw] OR Team sports[mesh])  AND  (Mood disorders[mesh] OR mood disorders[tw] OR Mental disorders[mesh] OR mental disorders[tw] OR Mental health[mesh] OR mental health[tw] OR Sleep wake disorders[mesh] OR sleep disturbance[tw] OR Substance-related disorders[mesh] OR Anxiety[mesh] OR anxiety[tw] OR Depression[mesh] OR depression[tw] OR Psychological distress[mesh] OR Chronic traumatic encephalopathy[mesh] OR alcohol use[tw] OR alcohol misuse[tw] OR alcohol abuse[tw] OR drug use[tw] OR drug misuse[tw] OR drug abuse[tw] OR substance use[tw] OR substance misuse[tw] OR substance abuse[tw])  NOT (("infant"[mesh] OR "child"[mesh] OR "adolescent"[mesh]) NOT "adult"[mesh])  NOT (animals[mesh] NOT humans[mesh])  NOT ("congress"[Publication Type] OR "editorial"[Publication Type] OR "letter"[Publication Type])  AND (English[language])  AND ("COVID-19" OR "COVID-19"[MeSH Terms] OR "COVID-19 Vaccines" OR "COVID-19 Vaccines"[MeSH Terms] OR "COVID-19 serotherapy" OR "COVID-19 serotherapy"[Supplementary Concept] OR "COVID-19 Nucleic Acid Testing" OR "covid-19 nucleic acid testing"[MeSH Terms] OR "COVID-19 Serological Testing" OR "covid-19 serological testing"[MeSH Terms] OR "COVID-19 Testing" OR "covid-19 testing"[MeSH Terms] OR "SARS-CoV-2" OR "sars-cov-2"[MeSH Terms] OR "Severe Acute Respiratory Syndrome Coronavirus 2" OR "NCOV" OR "2019 NCOV" OR (("coronavirus"[MeSH Terms] OR "coronavirus" OR "COV") AND 2019/11/01[PDAT] : 3000/12/31[PDAT])) |
| **EMBASE (March 31^st^ 2024)** |
| (sex/exp OR sex OR "sex differences" OR Male/exp OR Male OR Female/exp OR Female:ti,ab,de,kw OR Man:ti,ab,de,kw OR men/exp OR men:ti,ab,de,kw OR Woman:ti,ab,de,kw OR women/exp OR women:ti,ab,de,kw OR gender:ti,ab,de,kw)  AND  (College:ti,ab,de,kw OR collegiate:ti,ab,de,kw OR university:ti,ab,de,kw  OR semi-professional:ti,ab,de,kw  OR professional:ti,ab,de,kw  OR "national team":ti,ab,de,kw  OR Olympic:ti,ab,de,kw  OR Olympics:ti,ab,de,kw OR elite:ti,ab,de,kw) AND (Athlete/exp OR Athletes:ti,ab,de,kw  OR Athlete:ti,ab,de,kw  OR Sport/exp OR sports:ti,ab,de,kw  OR sport:ti,ab,de,kw  OR "Team sport"/exp) AND ("Mood disorder"/exp OR "mood disorders":ti,ab,de,kw  OR "mental disorders":ti,ab,de,kw  OR "Mental health"/exp OR "mental health":ti,ab,de,kw  OR "Sleep disorder"/exp OR "sleep disturbance":ti,ab,de,kw OR 'drug dependence'/exp OR Anxiety/exp OR anxiety:ti,ab,de,kw  OR Depression/exp OR depression:ti,ab,de,kw  OR 'distress syndrome'/exp OR "Chronic traumatic encephalopathy"/exp OR "alcohol use":ti,ab,de,kw  OR "alcohol misuse":ti,ab,de,kw OR "alcohol abuse":ti,ab,de,kw  OR "drug use":ti,ab,de,kw OR "drug misuse":ti,ab,de,kw  OR "drug abuse":ti,ab,de,kw  OR "substance use":ti,ab,de,kw OR "substance misuse":ti,ab,de,kw OR "substance abuse":ti,ab,de,kw) AND  ('covid-19' OR 'coronavirus disease 2019'/exp OR 'covid-19 vaccines' OR 'sars-cov-2 vaccine'/exp OR 'covid-19 serotherapy' OR 'covid-19 nucleic acid testing' OR 'covid-19 nucleic acid testing'/exp OR 'covid-19 serological testing' OR 'covid-19 serological testing'/exp OR 'covid-19 testing' OR 'covid-19 testing'/exp OR 'sars cov 2' OR 'severe acute respiratory syndrome coronavirus 2'/exp OR 'severe acute respiratory syndrome coronavirus 2' OR ncov OR '2019 ncov' OR 'coronavirus infection'/exp OR coronavirus OR cov)  AND ([adult]/lim OR [aged]/lim OR [middle aged]/lim OR [very elderly]/lim OR [young adult]/lim) AND [humans]/lim  NOT ('conference abstract'/it OR 'conference paper'/it OR 'conference review'/it OR 'editorial'/it OR 'letter'/it)  AND [english]/lim  AND [01-11-2019]/sd |
| **Cochrane CENTRAL (March 31^st^ 2024)** |
| [mh sex] OR sex:ti,ab,kw OR "sex differences":ti,ab,kw OR [mh Male] OR Male:ti,ab,kw OR [mh Female] OR Female:ti,ab,kw OR Man:ti,ab,kw OR [mh men] OR men:ti,ab,kw OR Woman:ti,ab,kw OR [mh women] OR women:ti,ab,kw OR gender:ti,ab,kw  AND  College:ti,ab,kw OR collegiate:ti,ab,kw OR university:ti,ab,kw OR semi professional:ti,ab,kw OR professional:ti,ab,kw OR "national team":ti,ab,kw OR Olympic:ti,ab,kw OR Olympics:ti,ab,kw OR elite:ti,ab,kw AND [mh Athletes] OR Athletes:ti,ab,kw OR Athlete:ti,ab,kw OR [mh Sports] OR sports:ti,ab,kw OR sport:ti,ab,kw OR [mh "Team sports"] AND [mh "Mood disorders"] OR "mood disorders":ti,ab,kw OR [mh "Mental disorders"] OR "mental disorders":ti,ab,kw OR [mh "Mental health"] OR "mental health":ti,ab,kw OR [mh "Sleep wake disorders"] OR "sleep disturbance":ti,ab,kw OR [mh "Substance-related disorders"] OR [mh Anxiety] OR anxiety:ti,ab,kw OR [mh Depression] OR depression:ti,ab,kw OR [mh "Psychological distress"] OR [mh "Chronic traumatic encephalopathy"] OR "alcohol use":ti,ab,kw OR "alcohol misuse":ti,ab,kw OR "alcohol abuse":ti,ab,kw OR "drug use":ti,ab,kw OR "drug misuse":ti,ab,kw OR "drug abuse":ti,ab,kw OR "substance use":ti,ab,kw OR "substance misuse":ti,ab,kw OR "substance abuse":ti,ab,kw  AND  (COVID-19 OR [mh COVID-19] OR "COVID-19 Vaccines" OR [mh “COVID-19 Vaccines”] OR "COVID-19 serotherapy" OR "COVID-19 Nucleic Acid Testing" OR [mh "covid-19 nucleic acid testing"] OR "COVID-19 Serological Testing" OR [mh "covid-19 serological testing"] OR "COVID-19 Testing" OR [mh "covid-19 testing"] OR SARS-CoV-2 OR [mh “sars-cov-2”] OR "Severe Acute Respiratory Syndrome Coronavirus 2" OR NCOV OR "2019 NCOV" OR [mh coronavirus] OR coronavirus OR COV)  NOT ([mh infant] OR [mh child] OR [mh adolescent]) NOT [mh adult]  NOT ([mh animals] NOT [mh humans])  NOT congress:pt OR editorial:pt OR letter:pt AND English with Cochrane Library publication date from Nov 2019 to present |
