# Supplementary material for: Exploring mental health symptoms in elite athletes during the COVID-19 pandemic: A systematic review and meta-analysis on sex differences
Source: PLoS One. 2025 Jan 16;20(1):e0314996. doi: 10.1371/journal.pone.0314996 (PMC11737678; doi:10.1371/journal.pone.0314996)
Supplement: S2 Table — (DOCX) [file pone.0314996.s003.docx]

**S2 table. Risk of Bias Assessment**

| Study | Item 1 | Item 2 | Item 3 | Item 4 | Item 5 | Item 6 | Item 7 | Item 8 | Item 9 | Total |
| --- | --- | --- | --- | --- | --- | --- | --- | --- | --- | --- |
| Boudreault 2022[23] | 0 | 0 | 1 | 1 | 0 | 0 | 0 | 0 | 0 | 2 |
| Buonsenso 2022 [24] | 0 | 0 | 0 | 1 | 0 | 0 | 0 | 0 | 0 | 1 |
| Davila-Torres 2021[25] | 0 | 0 | 0 | 0 | 0 | 0 | 0 | 0 | 0 | 0 |
| di Cagno 2020 [26] | 0 | 0 | 0 | 1 | 0 | 0 | 0 | 0 | 0 | 1 |
| Fiorilli 2021[27] | 0 | 0 | 0 | 1 | 0 | 0 | 0 | 0 | 0 | 1 |
| Gouttebarge 2022 [9] | 0 | 0 | 0 | 1 | 0 | 0 | 0 | 0 | 0 | 1 |
| Guillot 2022[28] | 0 | 0 | 0 | 0 | 0 | 0 | 0 | 0 | 0 | 0 |
| Hakansson 2020[13] | 0 | 0 | 1 | 1 | 0 | 0 | 0 | 0 | 0 | 2 |
| Imboden 2021[29] | 0 | 0 | 0 | 0 | 0 | 0 | 1 | 0 | 0 | 1 |
| Ivarsson 2021[30] | 0 | 0 | 0 | 1 | 0 | 0 | 0 | 0 | 0 | 1 |
| Knowles 2021[31] | 0 | 0 | 1 | 1 | 0 | 0 | 0 | 0 | 0 | 2 |
| McLellan 2022[32] | 0 | 0 | 0 | 1 | 0 | 0 | 0 | 0 | 0 | 1 |
| Melone 2022[33] | 0 | 0 | 1 | 0 | 0 | 0 | 0 | 0 | 0 | 1 |
| ParmÜ 2021[34] | 0 | 0 | 1 | 1 | 0 | 0 | 0 | 0 | 0 | 2 |
| Pensgaard 2021[35] | 0 | 0 | 0 | 1 | 0 | 0 | 0 | 0 | 0 | 1 |
| Pillay 2020 [36] | 0 | 0 | 0 | 0 | 0 | 0 | 0 | 0 | 0 | 0 |
| Salles 2022 [37] | 0 | 0 | 1 | 0 | 0 | 0 | 0 | 0 | 0 | 1 |
| Soares 2021 [38] | 0 | 0 | 0 | 0 | 0 | 0 | 0 | 0 | 0 | 0 |
