# Supplementary material for: Exploring mental health symptoms in elite athletes during the COVID-19 pandemic: A systematic review and meta-analysis on sex differences
Source: PLoS One. 2025 Jan 16;20(1):e0314996. doi: 10.1371/journal.pone.0314996 (PMC11737678; doi:10.1371/journal.pone.0314996)
Supplement: Attachment — Submitted filename: renamed_ddeb7.docx [file pone.0314996.s006.docx]

**Dear Dr. Kentiba:**

Thank you for the nice and detailed review. We have provided a **point-by-point revision** to all of your comments. The reasons and revisions are provided in the following tables. In the revised manuscript, all the changes are highlighted in **red.** We deeply appreciate your valuable feedback, which improved the quality of our submission. Thank you.

Sincerely

**Daphne Ling**

**September 29, 2024**

**Editor**

| **Opinion** | **Reply** |
| --- | --- |
| A numbered table of all studies identified in the literature search, including those that were excluded from the analyses.  For every excluded study, the table should list the reason(s) for exclusion.  If any of the included studies are unpublished, include a link (URL) to the primary source or detailed information about how the content can be accessed.  A table of all data extracted from the primary research sources for the systematic review and/or meta-analysis. The table must include the following information for each study:  Name of data extractors and date of data extraction  Confirmation that the study was eligible to be included in the review.  All data extracted from each study for the reported systematic review and/or meta-analysis that would be needed to replicate your analyses.  If data or supporting information were obtained from another source (e.g. correspondence with the author of the original research article), please provide the source of data and dates on which the data/information were obtained by your research group.  If applicable for your analysis, a table showing the completed risk of bias and quality/certainty assessments for each study or outcome. Please ensure this is provided for each domain or parameter assessed. For example, if you used the Cochrane risk-of-bias tool for randomized trials, provide answers to each of the signalling questions for each study. If you used GRADE to assess certainty of evidence, provide judgements about each of the quality of evidence factor. This should be provided for each outcome.  An explanation of how missing data were handled.  This information can be included in the main text, supplementary information, or relevant data repository. Please note that providing these underlying data is a requirement for publication in this journal, and if these data are not provided your manuscript might be rejected. | Response:  We have prepared this manuscript in line with the PRISMA Statement. We have included a PRISMA flowchart with the number of citations included in the systematic review process and their reasons for exclusion. Table 1 consists of the list of included studies, the study details, as well as the sample size for each study. The supplementary materials include the exact search string for each database and a risk of bias assessment for each study. The raw data for this meta-analysis are available upon request, and we have added this statement to the text. |

**Reviewer #1**

| **Opinion** | **Reply** |
| --- | --- |
| A competent and clinically relevant systematic review and meta-analysis on sex differences in mental health symptoms in elite athletes during the COVID-19 Pandemic. This will be of specific interest to sports medicine. | Response:  Thank you for your appreciation of this work. |

**Reviewer #2**

| **Opinion** | **Reply** |
| --- | --- |
| Please consider the following points: |  |
| Comment 1  Check key words in the abstract based on the Mesh standard. | Response 1:  Thank you for your comments. We checked the Mesh standard and modified the keywords as follows:  **Line 50**  Keywords: athletes; elite athletes; depression; anxiety; COVID-19; mental health |
| Comment 2:  Update the relevant reference about mental health of athletes during the pandemic. the following references are recommended:  Rassolnia, Atie, and Hadi Nobari. "The impact of socio-economic status and physical activity on psychological well-being and sleep quality among college students during the COVID-19 pandemic." International Journal of Sport Studies for Health 7.2 (2024): 1-12.  Taheri, Morteza, et al. "Mental health, eating habits and physical activity levels of elite Iranian athletes during the COVID-19 pandemic." Science & Sports 38.5-6 (2023): 527-533.  Taheri, Morteza, et al. "Comparative study of the long-term impact of the COVID-19 pandemic on mental health and nutritional practices among international elite and sub-elite athletes: a sample of 1420 participants from 14 countries." Sports medicine-open 9.1 (2023): 104. | Response 2:  Thanks for providing these additional references. We noted the two references (Taheri 2023a, Taheri 2023b) in the initial search, and excluded them with reasons (lack of gender-specific data). The reference Rassolnia et al. is excluded because the population does not consist of athletes. |
| Comment 3  The reliance on a wide range of assessment tools (such as the GAD-7, PHQ-9, etc.) adds heterogeneity to the results, which could limit the comparability of findings across studies. While the authors acknowledge this, a more uniform approach or further subgroup analysis might have strengthened the results. | Response 3:  Thanks for your great comments. We agree that a wide range of assessment tools adds heterogeneity to the results, limiting the comparability of findings across studies. Many tools have been validated for one mental health symptom, and the variety that is found in the literature precluded a more uniform approach. Subgroup analysis may be helpful, but was prevented by the limited number of studies reporting the outcome using the same tool. We addressed this point in the limitations section.  **Line 285**  First, a major limitation was the variability of scales used by the included studies to assess each mental health symptom, which could contribute to the substantial heterogeneity observed in our results. Furthermore, certain mental health symptoms featured a limited number of studies, thereby prohibiting further meta-analysis and subgroup analysis to investigate potential sources of heterogeneity, such as geographic region or sport type. |
| Comment 4  The use of a random-effects model is appropriate for accounting for inter-study variability. However, the authors mention significant heterogeneity in some outcomes but do not explore potential sources of heterogeneity in depth, such as geographic region or sport type (team vs. individual sports). | Response 4:  Thank you for this comment. We agree that exploring heterogeneity may be necessary for some factors. Subgroup analysis may be helpful, but was prevented by the limited number of studies reporting the same factors. We addressed this point in the limitations section.  **Line 285**  First, a major limitation was the variability of scales used by the included studies to assess each mental health symptom, which could contribute to the substantial heterogeneity observed in our results. Furthermore, certain mental health symptoms featured a limited number of studies, thereby prohibiting further meta-analysis and subgroup analysis to investigate potential sources of heterogeneity, such as geographic region or sport type. Second, our study was limited by data unavailability for specific comparisons. Stratifying our analysis based on team versus individual sports was not possible, as most studies incorporated both types. |
| Comment 5  The findings—female athletes reporting higher levels of anxiety, depression, and distress—align with broader mental health trends, yet the causal interpretations are limited due to the cross-sectional nature of most studies. whats your justification? | Response 5:  Thanks for your comments. We agree that the causal interpretations are limited in this SR due to the cross-sectional nature of most studies. We did address this point in the section on limitations:  **Line 296**  An additional limitation pertains to the cross-sectional design of nearly all of the included studies. We could not evaluate the progression of these mental health symptoms at various stages of the pandemic lockdown, nor could we determine if gender differences in these mental health symptoms persisted as the pandemic progressed. A meta-analysis of longitudinal cohort studies in the non-athletic population comparing mental health before and during the pandemic revealed a slight increase in mental health symptoms shortly after the COVID-19 pandemic began, which then decreased and became comparable to pre-pandemic levels by mid-2020 among most populations [59]. This trend may be attributed to effective resilience and adaptation; however, significant heterogeneity existed among subgroups, and time-lag effects may also be present [60] |
| Comment 6  The discussion effectively highlights the unique challenges faced by female athletes, including financial and career uncertainties. However, the article could have offered more concrete recommendations for sports organizations on how to address these issues, especially in light of the highlighted mental health disparities. | Response 6:  Thanks for your great comments. We noted the unique challenges faced by female athletes, including financial and career uncertainties, and gave recommendation for sports organization in the conclusions.  **Line 312**  It is crucial to address this sex-based disparity by providing tailored interventions and support strategies to mitigate the negative impact on female athletes' mental health and performance during major disruptive events such as a pandemic. Diligent observation and assessment of athletes' mental health is a vital component of any sport and should incorporate mental health resources such as mindfulness-based initiatives, stress management, or resilience assistance [18].  **Reference:**  Reardon CL, Hainline B, Aron CM, Baron D, Baum AL, Bindra A, et al. Mental health in elite athletes: International Olympic Committee consensus statement (2019). Br J Sports Med. 2019;53(11):667-99. |

**Reviewer #3**

|  | **Reply** |
| --- | --- |
| The authors investigated the sex differences of the impact of the C19 pandemic in elite athletes through a systematic review and meta-analysis. The results showed that female athletes reacted differently to the C19 lockdowns with higher levels of anxiety, depression and stress. However, this didn’t have any influence on their alcohol use, nor eating disorders, gambling, substance use or sleep problems.  Clear and concise systematic review with meta-analysis. | Response:  Thank you for your positive feedback. |
| Comment 1  L234. Maybe mention that alcohol use was different in one study (Imboden et al.) of this meta-analysis. | Response 1:  Thanks for your comment. We have added this point to the corresponding line:  **Line 253**  No differences were found in alcohol misuse between elite male and female athletes in the meta-analysis. Only Imboden et al. showed that male sex is a well-known risk factor for increased alcohol use in the general population [29]. |
